# Neonatal perforated appendicitis. Case report

**DOI:** 10.1016/j.ijscr.2024.110748

**Published:** 2024-12-16

**Authors:** Diego Herrera Ojeda, Esperanza Vidales-Nieto, Antonio Medina Vega, Victoria Damián Cuellar, Horacio G. Carvajal, Arturo Javier Cavazos Castro

**Affiliations:** a Department of Pediatric Surgery, National Institute of Pediatrics, Av. Insurgentes Sur No. 3700-C, 04530 Mexico City, Mexico; b Division of Pediatric Cardiothoracic Surgery, University of Utah, United States of America

**Keywords:** Neonatal appendicitis, Pediatric, Case report

## Abstract

**Introduction and importance:**

Neonatal appendicitis is a rare condition with high morbidity and mortality due to its late diagnosis in favor of more common pathologies. There are few reported cases of neonatal appendicitis and even fewer of antenatal appendicitis.

**Case presentation:**

We report a neonate presenting with abdominal distention and gastric emesis in the setting of a suspected congenital abdominal mass, later diagnosed with neonatal appendicitis requiring intestinal resection and anastomosis.

**Clinical discussion:**

Neonatal appendicitis presents with nonspecific symptoms and may often be mistaken for enterocolitis or intestinal malrotation. Additional imaging, including abdominal radiographs, ultrasound, or computed tomography, may facilitate diagnosis.

**Conclusion:**

Neonatal appendicitis is a rare entity with a challenging diagnosis due to the lack of specific signs. In the current case, the patient was referred to our institution with an antenatal ultrasound showing a right flank mass which resulted in abscess formation and perforated appendicitis.

## Highlights

• Neonatal appendicitis is a rare condition with high mortality due to delayed diagnosis.
• Increased risk of perforation due to anatomic factors, resulting in greater morbidity
• May occur with cystic fibrosis or Hirschsprung disease, meriting further work-up
• Nonspecific symptoms may be confused with NEC or malrotation.
• Radiographs, ultrasound, and computed tomography may help in the diagnosis.

## 1 Introduction

Neonatal appendicitis is extremely rare, with an incidence between 0.05 and 0.2 %. Most cases remain undiagnosed until laparotomy, leading to an increased rate of perforation, peritonitis and high mortality [[1], [2], [3], [4], [5]]. There are fewer than 200 reported cases of appendicitis in the neonatal period, with antenatal diagnosis being even more infrequent [6,7].

We report a case of neonatal appendicitis in an 8-day-old newborn who was referred to our institution with a prenatal ultrasound showing a right flank mass suspicious for a hepatic tumor. The patient underwent surgery on the third day of hospitalization. Intraoperative findings were notable for a perforated appendix and right flank abscess, as well as inflammation and a serosal tear in the ascending colon, requiring bowel resection and anastomosis.

## 2 Methods

This case report has been reported in line with the SCARE criteria [8].

## 3 Case report

**Fig. 1:**
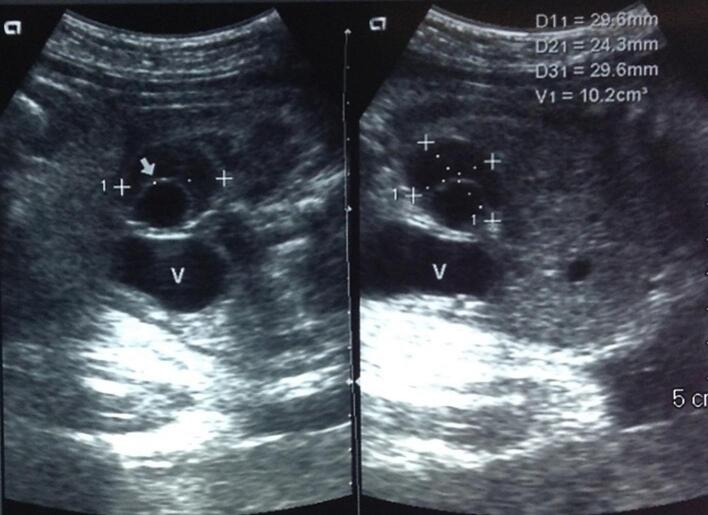
Abdominal ultrasound showing a right upper quadrant hypoechoic mass with an internal hyperechoic rim.

An eight-day-old newborn born via spontaneous vaginal delivery with a birth weight of 3610 g was referred to our institution for workup of an intra-abdominal mass. Prenatal ultrasound performed at 32 weeks gestation showed a 2.9 × 2.4 × 2.9 cm cystic mass in the right upper quadrant, suspicious for a hepatic lesion (Fig. 1).

Prior to admission, she presented with gastric emesis and mild abdominal distension. On exam, she was afebrile, with a soft, non-tender abdomen, and no palpable masses. Her laboratory exams revealed hemoglobin 12 mg/dL, hematocrit 36 %, WBC 11.4 × 10^9^/L, platelets 295 × 10^9^/L, aspartate aminotransferase 13 IU/L, alanine aminotransferase 8 IU/L, gamma glutamyl transpeptidase 50 IU/L, alkaline phosphatase 92 IU/L, lactate dehydrogenase 260 IU/L and alpha-fetoprotein 14,919 IU/L.

**Fig. 2:**
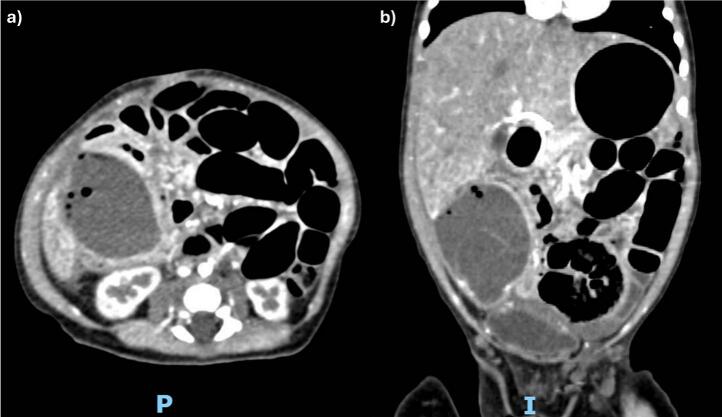
Axial (a) and coronal (b) abdominal CT scan with intravenous contrast showing a right-sided heterogeneous abdominal mass filled with air and liquid, adjacent to the liver and small bowel.

Abdominal radiograph showed no distended loops of small bowel, signs of pneumoperitoneum or pneumatosis, while the ultrasound showed a 4.6 × 3.3 × 4.6 cm non-mobile, cystic mass containing fluid and air, with peripheral vascularity (Fig. 1). Given these findings, we performed a contrast-enhanced computed tomography (CT) scan, which showed a right-sided abdominal mass filled with air and liquid (Fig. 2), suspicious of an intestinal duplication rather than a liver mass. Given the patient's imaging findings, we elected to perform an elective laparotomy via midline incision.

**Fig. 3:**
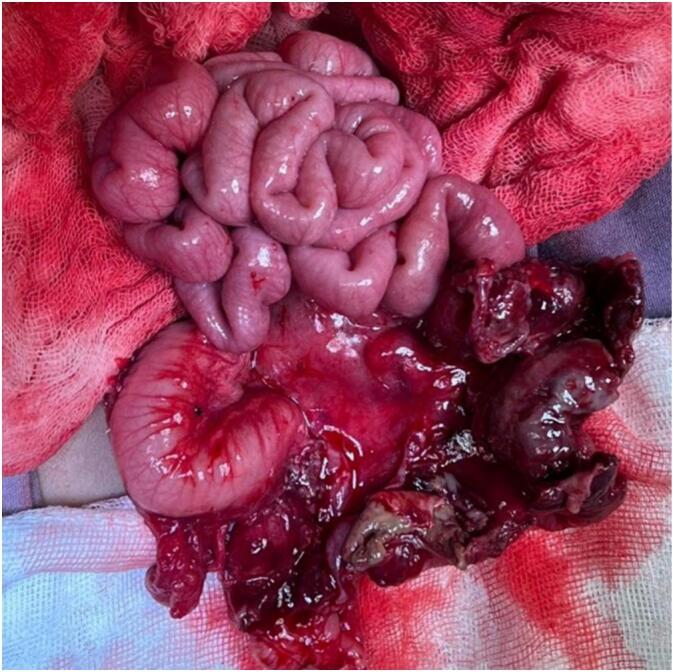
Gross examination of the intestine, with the distal ileum and ascending colon with a large perforation involving the base of the appendix.

Upon entry to the abdomen, we found multiple dilated, fluid-filled bowel loops, as well as tight adhesions between the ascending colon and the abdominal wall. While dissecting these, we encountered a 2x2cm mass, previously seen on abdominal ultrasound, which released purulent drainage on further dissection. The distal ileum and the ascending colon were hyperemic, with a serosal tear and large perforation involving the base of the appendix (Fig. 3). Due to these findings, we performed a distal ileocecectomy with primary end-to-end ileo-colonic anastomosis.

**Fig. 4:**
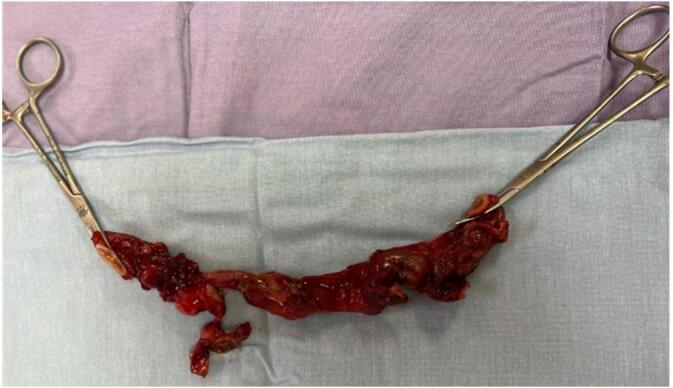
Resected segment including 10 cm distal ileum and the ascending colon with the cecum and perforated appendix.

Gross examination of the specimen revealed a 14.5 cm-long segment of bowel with an attached 3.6 cm-long appendix (Fig. 4). The appendiceal base was grossly hemorrhagic and perforated. Microscopic examination of the appendix demonstrated acute and chronic inflammation; the rest of the resected bowel similarly showed signs of inflammation, with no evidence of perforation or necrosis.

Following surgery, she was kept NPO and supported with total parenteral nutrition and antibiotics for five days. On postoperative day 5, she was tolerating an oral diet of breast milk and was subsequently discharged with no complications. At her latest follow-up visit, the patient was doing well, with appropriate weight and height for her age.

## 4 Discussion

The present case of an infant presenting with non-specific symptoms, including abdominal distension and gastric emesis, and abdominal imaging showing a heterogeneous right lower quadrant mass in the setting of antenatal perforated appendicitis highlights the diagnostic complexity of this pathology. On arrival to our institution, the patient was afebrile with a benign abdominal exam. The absence of traditional signs and symptoms, including leukocytosis, fever, and peritonitis on abdominal exam, likely resulted from the contained nature of the purulence within the patient's intra-abdominal abscess, further complicating the diagnosis. The rarity of neonatal appendicitis additionally placed this diagnosis low within our differential.

Neonatal appendicitis is a rare condition with an incidence of 0.05–0.2 %, with a perforation rate between 80 and 85 % and mortality rate as high as 28 % due to late diagnosis. There is a slight male predominance, and is more frequent in premature neonates [1,2,7]. The first case of neonatal appendicitis was reported in 1905 by Albrecht, while the first suspicious case of antenatal appendicitis was reported in 1925 by Hill [1,9]. Additional reports of suspected antenatal appendicitis found intraoperative calcified lesions and cystic masses [[10], [11], [12], [13]]. The low incidence of neonatal appendicitis is due to several protective factors against appendiceal luminal obstruction, including a funnel shape with a wide opening into the cecum, neonate's recumbent position, a liquid diet comprised of breast milk or formula (resulting in an absence of fecaliths), and low incidence of lymphatic hyperplasia due to gastrointestinal and respiratory infections [[1], [2], [3],^6^,^14^]. In addition to the late diagnosis, neonatal appendicitis has a high perforation rate due to anatomic factors, including a thin appendiceal wall, under-developed omentum, and non-distensible cecum [1,2,6]. Though mortality has decreased significantly, falling from a high of nearly 80 % at the beginning of the 20th century to 23 % between 1990 and 2014, it still remains much higher than the reported mortality rate of 0.04 % among older pediatric patients with complicated appendicitis [1,6,7,14]. The increased mortality in these patients is multifactorial; the lack of traditional symptoms results in a late diagnosis and intervention, which compounded with the previously mentioned anatomic risk factors result in an increased rate of perforation in a smaller abdominal cavity, allowing for diffuse contamination and exacerbating bowel injury, leading to septic shock [1,2,14].

The pathophysiology of neonatal appendicitis is uncertain, though three main theories have gained acceptance. The first posits neonatal appendicitis to represent a localized form of necrotizing enterocolitis (NEC) resulting in localized appendiceal inflammation due to impaired immunity, though the initial conservative treatment of NEC may in these instances further postpone surgery and definitive diagnosis [14]. The second theory states that neonatal appendicitis may result from vascular insufficiency or intestinal ischemia, such as in the case of an incarcerated Amyand hernia, leading to appendiceal strangulation and perforation [14]. This pathophysiology can also be seen in patients with cardiac anomalies, perinatal asphyxia, extracorporeal membrane oxygenation dependence, or hypoxic states [14,15]. Per the last theory, neonatal appendicitis is secondary to a cecal obstruction causing distention, as in the case of Hirschsprung disease or meconium ileus, leading to increased pressure at the base of the appendix and subsequent perforation [1,3,7,16]. Jancelewicz et al. reviewed published cases of neonatal appendicitis into three groups by etiology: impaired immunity or systemic infection, vascular insufficiency or hypoxia, and intestinal obstruction [16]. The majority of reports were associated with impaired immunity, including 39 instances of prematurity, or intestinal obstruction, with 30 instances of Amyand hernia and 6 cases of Hirschsprung's disease; only 5 were attributable to vascular insufficiency or hypoxia [16]. Histological diagnosis and evaluation of the appendix is important to diagnose and rule out diverse causes of this perforation, which may require further treatment [17]. Given that neonatal appendicitis can be the first sign of an illness underlying illness such as cystic fibrosis or Hirschsprung disease, additional evaluation, such as suction rectal biopsies, may be warranted as the appendix itself is aganglionic [2,6]. In the case of our patient, the underlying cause remains uncertain, though a suction rectal biopsy was negative for Hirschsprung's and he did not have evidence of cystic fibrosis or further symptoms.

Most neonates with appendicitis present with nonspecific signs and symptoms, such as abdominal distension and tenderness, vomiting, fever, malaise, irritability, leukocytosis, or intolerance to feeds. Physical examination is often unrevealing, limited to abdominal induration and right lower quadrant abdominal mass, making the diagnosis very difficult. In neonates, these symptoms are easily mistaken for NEC or gastroenteritis, which are initially managed conservatively with antibiotics, fasting, and serial abdominal exams in the absence of hemodynamic instability [1,3,6,16]. In the case of neonatal appendicitis, however, this approach would lead to an avoidable delay in definitive surgical treatment. To avoid this, Schwartz et al. proposed a diagnostic algorithm for neonates with abdominal distension and sepsis, where those with abdominal x-ray evidence of perforation or obstruction would immediately go for exploratory laparoscopy or laparotomy, while those with evidence of NEC, such as pneumatosis intestinalis, would undergo targeted treatment for this pathology [1,6]. In the instances where abdominal x-ray shows no evidence of obstruction, perforation, or NEC, their algorithm suggests further imaging with abdominal ultrasound with Doppler, allowing for evaluation of the bowel and its perfusion [6]. The authors posit that the benefits of early laparotomy in the absence ultrasound findings suggestive of NEC or definitive appendicitis may outweigh the risks and lead to improved survival [6]. Although CT tends to be used scarcely in neonates due to the significant radiation exposure, urgent abdominal CT with contrast is may show specific signs of appendicitis, such as periappendiceal fat stranding, an inflamed appendix, intra-abdominal abscess, or calcification, leading to surgical exploration for definitive management when unable to obtain abdominal Doppler ultrasound or when the ultrasound findings are equivocal [2,6,10,18]. Due to the rarity of this pathology and the high mortality, the potential benefits of a computed tomography an early surgery may outweigh the risk of cancer associated with radiation exposure from CT [6]. In the case we present, abdominal x-ray was negative for NEC or malrotation, while both abdominal ultrasound and CT showed a large mass, which was found intraoperatively to be an abscess in the setting of perforated appendicitis. Though not specific for antenatal appendicitis, findings such as the cystic intra-abdominal mass found on this patient's gestational ultrasound are suggestive of this pathology [1,6].

Though the most common surgical approach for neonatal appendicitis is via exploratory laparotomy, several reports of laparoscopic management have been published as well. This approach provides several advantages, such as decreased morbidity and a less-invasive surgery [19]. This approach also significantly shortens the postoperative recovery period and may reduce the risk of subsequent adhesions, without precluding conversion to open laparotomy if needed [19]. Although there is limited evidence in this age group, the potential advantages of laparoscopy in neonates make this a reasonable approach.

## 5 Conclusion

In conclusion, neonatal appendicitis is a rare cause of abdominal pain and distension in neonates which pose a significant challenge to prompt diagnosis. These patients are at increased risk of perforation and death compared to their older counterparts. This case illustrates a case of a neonate with antenatal imaging of an abscess secondary to a perforated appendicitis, requiring exploratory laparotomy and distal ileocecectomy with ileocolonic anastomosis.

## Authorship

All authors attest that they meet the current ICMJE criteria for authorship.

## CRediT authorship contribution statement

Diego Herrera Ojeda – Data collection and analysis
Arturo Cavazos Castro – Data collection, analysis and writing paper.
Esperanza Vidales-Nieto – Study concept
Francisco Antonio Medina Vega – Study concept, interpretation
Victoria Damián Cuellar – Data collection and analysis
Horacio G. Carvajal – Analysis and writing paper.

## Informed consent

Parental consent for minors: Written informed consent was obtained from the patient's parents/legal guardian for publication and any accompanying images. A copy of the written consent is available for review by the Editor-in-Chief of this journal on request.

## Ethical approval

This case report is exempt from ethical approval by our institution. The ethics committee at our institution (Instituto Nacional de Pediatría), does not review case reports that do not involve prospective intervention or significant risk to the patient. Due to this case report is a retrospective, single case with anonymized data, it was not considered to be reviewed.

## Guarantor

Arturo Javier Cavazos Castro is the guarantor of the work.

## Funding

This research did not receive any specific grants from funding agencies in the public, commercial, or non-profit sectors.

## Declaration of competing interest

The authors declare that they have no competing economic interests or known personal relationships that could have influenced the work reported in this document.
